# Association between polygenic risk scores for psychiatric disorders and social cognition: a systematic review

**DOI:** 10.1192/j.eurpsy.2022.2262

**Published:** 2022-09-01

**Authors:** M. Martinez, C. Fichera, L. Fusar-Poli, A. Rodolico, S. Guloksuz, E. Aguglia, M.S. Signorelli

**Affiliations:** 1 University of Catania, Department Of Clinical And Experimental Medicine, Catania, Italy; 2 Maastricht University, Dept. Psychiatry And Neuropsychology, Maastricht, Netherlands

**Keywords:** social cognition, autism spectum disorder, Psychosis, Polygenic Risk Score

## Abstract

**Introduction:**

Social cognition refers to a complex set of mental abilities that support the construction of adequate social competence and adaptation. Impairments in social cognition can be found in several psychiatric disorders, particularly in psychoses. Polygenic Risk Scores (PRSs) represent single metrics of molecular genetic risk and are a predictor of the genetic susceptibility to diseases, although they explain only a small part of the risk.

**Objectives:**

To explore the association between PRS for psychiatric disorders and social cognition.

**Methods:**

We conducted a systematic search in PubMed and Scopus according to the PRISMA guidelines up to August 2021. We included papers evaluating PRS and social cognition with psychometric scales. Articles concerning single-nucleotide polymorphisms and biological measures of social cognition (e.g., neuroimaging, peripheral biomarkers) were excluded.

**Results:**

We initially retrieved 150 articles. After removing duplicates, we screened 133 titles and abstracts and preliminary selected 19 papers. Participants recruited in the papers of interest were either people with schizophrenia, ASD or ADHD, their family members or healthy subjects. Articles evaluated the association between different psychometrical measures of social cognition and PRS for schizophrenia, Autism Spectrum Disorder and ADHD.

**Conclusions:**

Literature regarding the association between PRS for psychiatric disorders and social cognition is heterogeneous in terms of populations, genetic risk evaluation, and outcome tools. Given the critical role played by social cognition in the onset and progression of mental disorders and its association with real-world functioning, future research should try to disentangle the complex genetic basis of this domain.

**Disclosure:**

No significant relationships.

